# Evidence use in equity focused health impact assessment: a realist evaluation

**DOI:** 10.1186/s12889-019-6534-6

**Published:** 2019-02-26

**Authors:** Ingrid Tyler, Bernie Pauly, Jenney Wang, Tobie Patterson, Ivy Bourgeault, Heather Manson

**Affiliations:** 1 Fraser Health Authority, Surrey, BC Canada; 2 School of Nursing, Scientist, Canadian Institute for Substance Use Research, UVIC Community Engaged Scholar , University of Victoria, Victoria, BC Canada; 3 University of Toronto, Toronto, ON Canada; 4 Telfer School of Management, the University of Ottawa, Canadian Institutes of Health Research Chair in Gender, Work and Health Human Resources, Ottawa, Canada; 5 Chronic Disease and Injury Prevention, Public Health Ontario, Toronto, Ontario Canada

**Keywords:** Health inequity, Equity engagement, Evidence use, Public health practice, Realist evaluation, Knowledge translation

## Abstract

**Background:**

Equity-focused health impact assessment (EFHIA) can function as a framework and tool that supports users to collate data, information, and evidence related to health equity in order to identify and mitigate the impact of a current or proposed initiative on health inequities. Despite education efforts in both the clinical and public health settings, practitioners have found implementation and the use of evidence in completing equity focussed assessment tools to be challenging.

**Methods:**

We conducted a realist evaluation of evidence use in EFHIA in three phases: 1) developing propositions informed by a literature scan, existing theoretical frameworks, and stakeholder engagement; 2) data collection at four case study sites using online surveys, semi-structured interviews, document analysis, and observation; and 3) a realist analysis and identification of context-mechanism-outcome patterns and demi-regularities.

**Results:**

We identified limited use of academic evidence in EFHIA with two explanatory demi-regularities: 1) participants were unable to “identify with” academic sources, acknowledging that evidence based practice and use of academic literature was valued in their organization, but seen as less likely to provide answers needed for practice and 2) use of academic evidence was not associated with a perceived “positive return on investment” of participant energy and time. However, we found that knowledge brokering at the local site can facilitate evidence familiarity and manageability, increase user confidence in using evidence, and increase the likelihood of evidence use in future work.

**Conclusions:**

The findings of this study provide a realist perspective on evidence use in practice, specifically for EFHIA. These findings can inform ongoing development and refinement of various knowledge translation interventions, particularly for practitioners delivering front-line public health services.

## Background

Health inequities are defined as systematic and potentially remediable differences in one or more aspects of health across socially, demographically, or geographically defined populations or population subgroups [1]. These differences are not only unnecessary and avoidable, but unfair and unjust [2]. In a comprehensive review of practices which contribute to reductions in health inequities, [3] the use of equity-focussed health impact assessment (EFHIA) was identified as one of ten promising practices. EFHIA provides a framework of analysis, with the user inputting evidence for the effective consideration of potential equity impacts. As a tool, EFHIA supports users to collate existing data, information, and evidence related to health equity in order to identify and mitigate the impact of a current or proposed initiative on health inequities. In this way, EFHIA supports knowledge uptake and utilization in practice.

Despite dissemination, training, and efforts at application in both the clinical and public health settings, practitioners have found implementation and the use of evidence in completing EFHIA tools to be challenging, demonstrating an important knowledge-to-action gap [4, 5]. Based on the first author’s experience, practitioners often request additional support to apply EFHIA in their local contexts, including support to translate the evidence needed to complete the tool. In some cases, completion of EFHIA is hindered by initial experiences, with inadequate guidance to identify, access, interpret, synthesize, and apply evidence into an EFHIA framework.

In this paper, we report on a realist evaluation of EFHIA completion conducted in four public health practice sites using a mixed methods case study approach. Realist science seeks to generate explanations through observing patterns in the data that recur often enough to support hypothesized mechanisms of action [6]. These explanations are articulated as Context-Mechanism-Outcome (CMO) configurations. Pawson et al., [5] indicate that to infer an outcome (O) between two events, one needs to understand the underlying mechanism (M) that connects them and the context (C) in which the relationship occurs (p.2). For the purposes of this research we define mechanisms as cognitive or emotional responses related to context that “turn on” the minds of program participants and stakeholders in such a way as to make them want to achieve the outcomes of the program [7]. In realist evaluation therefore, the question shifts from ‘what works’ to ‘what is it about this programme that works for whom in what circumstances?’ (p. 2) [5].

## Methods

Realistic evaluation is an emerging methodological approach [8], as indicated by a handful of published studies using this approach [9–12]. Using realist evaluation, we sought to understand the use of evidence in the EFHIA process to inform specific knowledge translation (KT) interventions that support application of evidence in decision support tools. Our overall objective was to understand how to reduce the observed gap in use of health equity evidence in completing health equity assessment tools.

The realist evaluation cycle consists of three main phases [13]:

1. Theory and proposition development
2. Observations through multi-method data collection
3. Analysis and identification of CMO configurations and demi-regularities

### Realist evaluation phase I: Theory and proposition development

Developing propositions was informed by a scan of the literature, stakeholder engagement, three existing theoretical frameworks, and abductive reasoning.

#### Literature scan

A total of 986 relevant abstracts were found through a librarian-guided search strategy, hand searching and expert advice. Articles covered realist evaluation, EFHIA evaluation, and/or evidence informed decision making in public health.

#### Stakeholder engagement

We conducted informal interviews with five knowledge users from each case study (public health unit) site and ten research team collaborators. Interviews sought to understand individual perspectives on the use of evidence in the EFHIA, the facilitators and barriers that practitioners experience in engaging with health equity evidence, and their definition of success in the use of evidence.

#### Theoretical frameworks

We were informed by three existing evidence-to-practice theoretical frameworks that best informed our research question. First, we drew on a KT review conducted by the Institut National de Santé Publique du Quebec (INSPQ) [14]. The INSPQ review takes into account organizational complexity and focuses on the continuous interaction between various groups of actors to reduce the gap between the world of research and practice. It describes six steps of the KT process: production, adaptation, dissemination, reception, adoption, and knowledge appropriation and use. It defines knowledge brokers as intermediaries facilitating the interaction between knowledge producers (academics, researchers) and decision makers (practitioners, policy makers). Second, we incorporated two dimensions of the Equity Knowledge Translation framework [15]: critical inquiry of knowledge and reflexive practice in knowledge translation. Critical inquiry of knowledge relates to how knowledge is valued and accepted, leading other knowledge bodies to being subordinated or ignored. Reflexive practice relates to one’s influence within systems, which involves a process of self-examination. These two concepts suggest that explanations for social inequalities in health relate to priorities and underlying ideologies that lead to organizational limitations or leanings that may obstruct health equity aims. Third, we drew on concepts from the National Collaborating Centre for Methods and Tools [16] definition of public health evidence. This includes evidence from published peer reviewed literature, evidence from surveillance and community health assessments, from clients and stakeholders about community preferences and actions, feasibility, human and financial resources and materials, and evidence from practice.

#### Abductive reasoning

Jagosh et al. (page 134, Table 1) [17] define abductive reasoning as: “inference to the best explanation. It involves an iterative process of examining evidence and developing hunches or ideas about the causal factors linked to that evidence”. We applied abductive reasoning in reviewing theoretical frameworks, evidence and stakeholder experiences, literature review results and stakeholder consultation, through informal concept mapping. This reasoning process was undertaken with reflexivity to ensure that the evaluation team would be able to assess whether or not the mechanism was in operation, paying close attention to the team’s ability to identify the contextual factors at play. The propositions below were developed from the concept map and validated with knowledge users and the research team.

**Table 1:** EFHIA Team Structures and Timelines

Location	EFHIA Team Profile	Topic Area
PHUA	3 front line staff; 1 to 12 years’ experience; previous experience with vulnerable populations; rare or occasional evidence experience	Oral Health - intended and unintended consequences of the promotional strategies
PHUB	4 front line staff; 11 to 35 years’ experience; previous experience with vulnerable populations; rare evidence experience	Sexual Health - to determine the impact of removing sexual health services from secondary schools
PHUC	5 managers; 15 to 34 years’ experience; 2 worked with vulnerable populations; 2 other health equity experience; rare or occasional use of evidence	Food Safety - the vendor application process
PHUD	2 policy staff/non-clinical; 4 to 25 years’ experience; worked with vulnerable populations; evidence experience high	Child Injury - health equity impact of injury prevention services provided to children in school settings and their parents in community settings

#### Initial propositions

In realist evaluation, the initial propositions are hypotheses that tie context, mechanism, and outcome together that are then tested and refined throughout the study [6]. Our initial propositions are:

- Knowledge brokering at the local site will facilitate evidence familiarity and manageability, and increase user **comfort and confidence** in processing the evidence.
- Involvement of users in the knowledge production process aligns evidence with user needs and increases **acceptance** of the information.
- Adapting the knowledge to match user characteristics can encourage evidence use because there is increased understanding of the knowledge and **consonance** with the content.
- **Correspondence** between knowledge produced and the problem to be solved can facilitate evidence use because the users will perceive the knowledge as applicable.
- Knowledge brokering during the knowledge production process can help build relationships with users, establish **trust** and familiarity in the producer, and facilitate evidence use.
- Knowledge brokering at the local site can facilitate evidence use because users have timely **access** to knowledge, reducing the perception of barriers.

### Realist evaluation phase II: Case study data collection

We used a multi-site, mixed methods case study, informed by Yin’s guide on case study research [18] to collect data on EFHIA tool completion at four case study sites. Case study methodology allows for an understanding of phenomena within a naturally occurring context, aligning well with tenets of realist evaluation which studies contextual differences in implementation [19]. Data collection methods focused on gaining information to understand context [C], establish outcomes [O] and identify underlying mechanisms [M], in order to test the initial propositions developed during phase I. Ethics approval was granted for this study from the Public Health Ontario Ethics Review Board and relevant ethics boards for each site.

#### Recruitment and sample

A convenience sample of five public health unit (PHU) case study sites already known to be implementing EFHIAs were approached to participate in the study. Four sites were recruited, and at each of these sites, a team of individuals actively involved in completing EFHIA formed the unit of our analysis. We refer to these as EFHIA teams. Each team used the EFHIA tool being implanted at their site, with some variation between tools. Case study sites included rural-urban and urban settings in two Canadian provinces (BC and Ontario). Topics covered by the EFHIA teams that were recruited to participate included oral health, food safety, sexual health, and child injury and prevention. The teams varied in size from two to five members, including front line staff members, managers, and policy analysts in varying combinations at each site. The time period that each team took to complete their health equity assessment tools ranged between four weeks and six months. Table 1 outlines the team structures and topics.

Separate from the members of the EFHIA team, key informants (KI) were also identified and recruited at each participating site. These were individuals involved in EFHIA implementation at the site, including key health unit leaders, community members, and staff who support health equity and evidence use in the organization. These individuals were identified as critical to understanding the EFHIA implementation and context specific to the case study site including the organization’s evidence culture and health equity culture.

### Case Study Data Collection and Analysis

Data collection methods included: 1) document review, 2) semi-structured interviews, 3) online surveys, and 4) observation (Table 2). Each data collection element focused on different aspects of context, mechanism or outcome as hypothesized in our initial propositions. The data collection instruments were developed based on instruments from the literature with a focus similar to our evaluation [20–24] as well as validated tools [25]. Data were analyzed using the most appropriate analytic tool (NVivo, Excel) and these data sources were triangulated to develop a comprehensive understanding of phenomena [26].

**Table 2:** Summary Table of Data Collection Activities

Activity	Baseline	Midpoint	End of Study
Document Review	✓		✓
KI Semi-structured Interviews	✓		✓
EFHIA Team Member Surveys	✓		✓
EFHIA Team Member Semi-structured Interviews		✓	✓
EFHIA Team Observation		✓	

#### Document review

For the baseline assessment, EFHIA team members and KIs were asked to provide documents such as organizational strategic and equity plans. At end of study, EFHIA teams were requested to submit their completed health equity assessment tools. These documents were read by the research team (IT, TP, JW) to provide additional context and outcome information. Relevant information was extracted into case study reports.

#### Semi-structured interviews

Key informants participated in two semi-structured interviews. They were interviewed at baseline to better understand organizational contexts, including equity and evidence culture, and policy landscapes. They were also interviewed at end of the study for information on the EFHIA use, outcomes and impact within the organization, as well as their experience with the quality of evidence use in the final products.

All individual EFHIA team members were interviewed at the midpoint in their EFHIA completion process and at end of study. Midpoint interview questions asked about team members’ experience with the tool completion process, with using and interpreting evidence, with any barriers and facilitators encountered, and about any anticipated outcomes from the tool. End of study interviews focused on the individual’s experience with completing an EFHIA, anticipated outcomes from EFHIA completion and any change in healthy equity knowledge or attitude.

All interviews were analyzed using NVivo 9 software. An initial codebook was created with subsequent thematic coding of interview data into categories. All coding was completed by researchers JW, TP and IT. The initial codebook was updated as new categories and subcategories emerged during analysis and until saturation was reached.

#### EFHIA team member surveys

Surveys were completed by EFHIA team members at baseline and end of study. Pre and post surveys included open and close-ended questions to assess EFHIA team members’ attitudes, values, motivations, levels of experience, educational and professional qualifications, previous experience with completing an EFHIA, mandate to support EFHIA, and perceptions of leadership attitudes towards EFHIA. The end of study survey contained follow-up questions to the baseline survey to enable comparison, as well as questions related to evidence use and outcomes.

Surveys were administered through SurveyMonkey and completed at the team members’ convenience within a specified timeframe. Online survey responses were exported into Excel and subsequently de-identified. For close-ended question responses (yes/no answers and Likert-scale), we used basic descriptive analysis to count the responses. Open-ended responses were transferred to NVivo and coded thematically.

#### EFHIA team observation

Lastly, we observed EFHIA teams during tool completion by observing meetings and reviewing relevant emails. The research team observed the EFHIA work processes, the KT activities taking place, barriers and facilitators encountered and any change in attitude that appeared to have occurred. Along with document review, team observation notes and email text were used to verify or interpret interview and survey data through the process described below.

Overall we had 14 EFHIA team participants complete the study over the four case study sites. A total of 11 and 9 of 14 EFHIA team members responded to the baseline and final surveys, respectively. We interviewed 15 key informants at baseline and 7 key informants at end of study (of which 3 had also participated in baseline interviews) and observed 15 team meetings.

### Realist evaluation phase III: Identification of context-mechanism-outcome configurations and Demi-regularities

Data were categorized according to context (C), mechanism (M) and outcome (O). Using case study analysis, processes of pattern matching, retroduction, and iteration for each of the outcomes were identified for each case study site. We then identified the mechanisms that were triggered to generate these outcomes, and the contexts within which these mechanisms were triggered. As the CMO configurations were developed, we sought confirmatory and contradictory evidence from all the site specific data available [27]. This CMO identification process was conducted for each case study site by individual researchers (IT, TP, JW) and through weekly case analysis meetings.

Once the CMOs operating at each site were identified, we tested our initial propositions through a process of pattern matching, [28, 29] which involves an attempt to link two patterns where one is a theoretical pattern and the other is observed. To the extent that the patterns matched, initial propositions were refined to support prediction of the observed patterns. Similar patterns across case study sites indicated stronger evidence for the proposition, while some propositions were corroborated by only one site experience.

After identifying patterns within and across study sites, we took a retroductive approach, hypothesizing possible mechanisms capable of generating the observed outcomes. Retroduction has been described as “abduction with a specific question in mind” [18] or “logic of inference” [6]. We established a clear documented chain of evidence, ensuring that in the development of these configurations, we took note of the evidence supporting each component of the CMO configuration as well as the links between them to enable cross-checking by other members of our research team. We created tables that displayed data from individual cases based on the outcomes of interest and the mechanisms initially informed by the initial propositions, gradually expanding to include newly identified CMO configurations [26]. In this way, analysis moved to cross-case analysis, by considering each case as a ‘whole study’ and then seeking convergent and contradictory evidence across cases [27, 30]. Through these inter-case comparisons, we identified semi-predictable patterns in the data - known as demi-regularities (coined by Lawson (cited in Jagosh et.al)) [31, 32].

The realist method of analysis is not linear, and involved an iterative analysis process with the sets of data outlined above [33]. We used a process of iteration between the intra-case and inter-case analyses until we developed study findings that answered the evaluation question. Certain case characteristics only became evident through inter-case comparisons, as context and other factors may influence mechanisms to lead to different outcomes in different cases.

We coded 1500 items over 300 categories in 15 themes. Not all case studies contributed to all categories. Each coding category had an average of two sites contributing data.

#### Member checking

Data from all sources were used to generate comprehensive accounts of EFHIA completion and evidence use in four different health unit contexts. Document review and team member observations were used to help interpret and triangulate data analysis and interpretation. Using all data sources, we generated case study reports for each site with observed study outcomes, and the CMO connections. These individual case study site reports were shared with research participants to ensure accuracy and resonance with their experiences. Participants were also contacted several months post study to participate in a data interpretation meeting over teleconference to verify if our demi-regularity interpretations of the data collected were accurately represented.

## Results

We present here the key findings from the CMO evidence for propositions and the two emerging demi-regularities.

### CMO evidence for propositions

Table 3 summarizes the CMOs identified at each site which were related to use of evidence. The majority of items were related to *contextual* categories, which included public health unit (PHU) description, team description, KT strategies, evidence and equity experience, attitudes towards evidence and equity, and EFHIA tool implementation. *Mechanism* categories included conceptual, practical and organizational factors. In total 14 potential mechanisms and 12 potential outcomes were identified from the data. *Outcome* categories included overall experience completing the tool, changes in attitude and use of evidence, engagement in the completion process, barriers and facilitators to evidence use, and actual evidence and equity engagement outcomes of tool completion. Not all context, mechanism or outcome categories identified could be matched to CMO relationships.

**Table 3:** Individual Case Study Site Context-Mechanism-Outcome Results

Case Study Site	Contextual factors	Mechanism^a^	Outcomes related to use of evidence
PHU A	Organizational support for and interaction with knowledge broker	Increased practitioner confidence; trust in knowledge broker	More likely to use academic evidence source in the future (self-report)
	Real or perceived lack of time or skill to interpret evidence	Unable to “identify with” academic sources; (i.e., lack of consonance)	Limited use of academic evidence (i.e., references) in EFHIA
	Familiarity with practical evidence (i.e., local surveillance, personal experience)	Safety and trust using own knowledge	Preferential use of practical evidence to complete EFHIA
PHU B	Set organizational direction for program action	Seeking alignment of evidence with desired action	Positive attitudinal change toward evidence (all sources)
	Real or perceived evidence characteristics of local data (reliable/relevant)	Evidence use experienced as positive “return on investment”; (i.e., good correspondence)	Use of practical evidence (i.e., local surveillance, personal experience)
	Real or perceived evidence characteristics of academic data (difficult to access/not directly relevant)	Evidence use experienced as negative “return on investment”; (i.e., poor correspondence)	Limited use of academic evidence
PHU C	Unclear expectations or unclear EFHIA mandate	Hesitancy or acquiescence to existing knowledge	Limited evidence sought
PHU D	Policy role in organization (non-clinical; non front-line service delivery oriented)	Strongly held individual and role-based evidence-use values	Increase use of academic evidence

^a^Mechanisms are cognitive or emotional responses related to context that “turn on” the minds of program participants and stakeholders in such a way as to make them want to achieve the outcomes of the program. [7]

**Proposition 1: Knowledge brokering at the local site can facilitate evidence familiarity and manageability, and increase user confidence in using evidence**

We found evidence supporting our initial proposition that knowledge brokering at the local site can facilitate evidence familiarity and manageability, and increase confidence in using the evidence.

*“In our case a big thing was having [name of knowledge broker] there …and we were more comfortable… in the fact that she could just help to shed a little bit of light, and then she was able to say, here’s a couple of places you might want to look for some information.” PHUA.*

Few respondents participating in the baseline survey had significant experience identifying (4/11), assessing (3/11), and incorporating (6/11) evidence directly into their decision making. Six of eleven practitioners (55%) responding to the baseline survey indicated a lack of confidence in incorporating public health evidence in their work.

While we did not see increased evidence use in this study, increased self-confidence encouraged participants’ intention to use more evidence in the future.

*“We’ll definitely be bringing it to the team …in future planning that we need to [be] taking into account adding some research aspect or literatures into any of the upcoming campaigns or projects that our team does.” PHUA.*

**Proposition 2: Evidence sources aligned with user needs increases acceptance and use of the information**

**Proposition 3: Adapting the knowledge to match user characteristics can encourage evidence use because there is increased understanding of the knowledge and consonance with the content**

**Proposition 4: Correspondence between knowledge produced and the problem to be solved can facilitate evidence use because the users will perceive the knowledge as applicable**

We were not able to corroborate our initial propositions as written; however we observed important trends in evidence use by type of information that correlated to our initial propositions 2, 3 and 4.

The most commonly accessed source of evidence used was local, practical evidence (including surveillance, grey literature and practice experience). This was the only evidence source majority of participants (10/11) stated during the baseline survey that they used “often” or “very often”. These sources were seen as accessible, available and applicable, and therefore more likely to be used. They were also considered to correspond better to user needs, providing practical information that could be applied directly to their work. In addition, we found that participants had more consonance, or identification with, sources that were familiar, including local surveillance data, PHU based data, and data from individual or others’ experience:

*“… the sources that I found useful have been conference presentations and poster sessions you will never see [in] a journal, right*? *So, you know, it really does kind of suggest that if you kind of want to find out what’s working in, local implementation of interventions to address inequity, you’re probably going to really have to make some connections with people at that level” PHUC.*

Another team member described referring to a health equity assessment tool completed by a team with a similar clinical background and feeling drawn to this evidence because *“we can relate to this and using their examples really helped us…just spark ideas or even go about filling [the tool] out, kind of got the ball rolling.”* *PHUB*.

There was one PHU team that demonstrated the opposite trend. The EFHIA team in PHUD “often” (2/2) used evidence from academic sources and rated the source highly. In this case, both team members were policy analysts as opposed to clinical staff or front line managers.

**Proposition 5: Knowledge brokering during the knowledge production process can help build relationships with users, establish trust and familiarity in the producer, and facilitate evidence use**

**Proposition 6: Knowledge brokering at the reception site can facilitate evidence use because users have timely access to knowledge, reducing the perception of barriers**

We observed that participants’ trust in the knowledge broker changed their attitude towards evidence and increased intention to use evidence in the future.

*“…the biggest help was having [knowledge broker] as kind of our navigator, … it was a little bit like a light bulb went off, and I thought, okay, I get this now. And then it started to be really rewarding, because we were coming up with ideas that we hadn’t thought of before…” PHUA.*

However, at one site we observed a different relationship with knowledge brokers, one of deference or “acquiescence”. In this case, participants were most likely to use evidence brought forward by the knowledge broker and simply accepted or deferred to sources that were easily available or brought to them rather than looking for evidence. Due to this acquiescence, other evidence sources were not explored. This occurred in a context of unclear mandate or expectations related to EFHIA.

*“I’ve relied on more of the committee, what has been brought to the table as compared to doing a ton of research on my own because I’ve got a number of other projects that are on my plate. So it’s sort of like I do read the stuff that comes in but I’m not going out and looking at it or evaluating it as I would assume that’s being done by those that are bringing it to the table.” PHUC.*

### Demi-regularities – Cross case conclusions

As described above, ‘demi-regularities’, are semi-predictable patterns where outcomes are linked to context through mechanisms [32, 34]. Our analysis identified two consistent demi-regularities tying context, mechanism and outcome together and providing explanatory insight into how evidence is used by practitioners in EFHIA.

**Demi-regularity 1: Practitioners are less likely to incorporate information that they do not understand or do not “identify with” (i.e., low or limited consonance with the information)**

Practitioners were unable to identify with academic sources, even while acknowledging that evidence based practice and use of academic literature was valued in their organization. Front line practitioners in particular acknowledged the importance of academic evidence “in theory” and evidence based practice as an organizational priority; however they had challenges appreciating their role in the evidence informed decision making process.

*“…I feel like the literature review was probably the most challenging, partly because it’s very foreign to us. As we’ve mentioned before, we don’t typically do literature reviews in our program, we’re very clinical people and it’s kind of out there for us....” PHUA.*

Additionally, as noted in the propositions above, EFHIA team members encountered challenges when interpreting academic literature, even when there was knowledge brokering and capacity building support available.

*“I must’ve read that article three times and I still didn’t really know if it was relevant at the end of it so I just went with it wasn’t relevant, right because… I didn’t really know what it was saying.” PHUB.*

**Demi-regularity 2: Practitioners will access information for decision making which is easily accessible, immediately available and directly applicable, for which they perceive a “positive return on investment”**

We identified across all health units that local evidence from practice (including surveillance, grey literature and practice experience) was associated with a perception of a “positive return on investment” (i.e., worth investing their energy and time).

*“I think that the population data and our internal statistics are really helpful ‘cause they say, hey, this is what we’re doing and this is who we’re meeting [the needs of] and who we’re not meeting [the needs of].” PHUC.*

In contrast, academic evidence was not seen as a good return on investment.

*“We did do a literature review as well but didn’t find a whole lot.” PHUA.*

*“…my opinion at the beginning [of] it would be that oh my gosh, there’s not enough time for all of this work and all of this collection of data to just make one decision.” PHUB.*

Team members felt a sense of comfort using local sources knowing that its relevance and applicability were sound.

*“…so it was kind of using our own sort of personal…clinical knowledge or clinical experiences that was the easiest to, kind of navigate through.” PHUA.*

## Discussion

We came to some important conclusions about the use of evidence in EFHIA in public health unit (PHU) case study sites in Canada. First and foremost, we did not observe much documented use of academic evidence, thereby highlighting the importance of knowledge translation (KT) research to support evidence based public health practice, particularly in the area of front-line health equity practice.

One of the significant observations found was differential use of evidence based on practitioner type. Most of our EFHIA case studies were assigned by local PHUs to front line practitioners for completion. While this makes sense, as they may be the most knowledgeable of their program, many of these staff did not feel that they had the experience to use formal evidence sources. While some had recent training in the area, these individuals still did not see this as an integral part of their job in which they should be updating their skills and competence. This included both staff and managers. In contrast, where we had policy level staff complete the tool, more evidence was used. This should lead us to question who is best positioned to complete the EFHIA tool within the public health structure – and identify other ways in which front line staff can best contribute to inform EFHIA completion.

We found the most common types of evidence used were surveillance data and personal practice experience. This is consistent with our initial proposition that consonance with the type and content of evidence and correspondence between knowledge produced and the problem to be solved are important mechanisms for evidence use. Again noting that the predominant practitioner type in our study was front line staff, it begs to reason that they were most comfortable with information that related directly to their practice experience and work, including local data, personal experience and grey literature/experience from nearby communities. Martin et al. [35] noted that practitioners “require evidence (or data) that is timely, relevant to their context and purpose, current and regularly updated, synthesized and translated into manageable bite sized pieces, trustworthy, and of different types at different levels.” Our research corroborated these findings as a “correspondence” mechanism.

McMahon [36] defined “consonance” as “an expression of the theme of ‘matching’ the intervention to the … participants’ … cultural values, norms and symbols to increase the symbolic understanding of interventions.” We interpret this as enabling practitioners to “identify with” the data source and content. To “identify with” the data is likely to mean different things to different types of practitioners. For local practitioners, this generally meant local sources. Many of our participants understood and acknowledged the importance of evidence-based practice, but felt that it took too much time in the context of their service delivery roles. This may also speak to a real lack of relevant and user driven public health evidence. Practitioners found academic literature difficult to incorporate, and were challenged to find sources that matched their local context and needs. They wanted immediate answers relevant to their local context and seemed to have challenges extrapolating more generalized results to their specific issues. This is a significant KT challenge. If users did not perceive a proportionate “return on investment” of information for the time and effort spent, they were less likely to try to access evidence in the future. The “return on investment” concept is not one that is common in the KT literature.

Knowledge brokers were able to help with evidence access and use. Many of our sites did not have formal knowledge brokering roles, and informal knowledge brokers emerged. There was often a high level of trust in the knowledge broker. Many EFHIA team participants seemed relieved to take the information presented to them by the knowledge broker and apply that to the program. Therefore it is imperative that if local health units were to implement a formal knowledge broker model that these individuals are well versed in critical appraisal, evidence interpretation, and options analysis, as their suggested sources or conclusions were not commonly questioned. There are some models for this that already exist, for example, National Collaborating Centre for Methods and Tools local knowledge broker mentoring program [37]. Launched in 2014, this program provides in-person and online support to train public health practitioners to develop knowledge and capacity in applying evidence informed decision making. Although no published evaluation of this program currently exists, anecdotal evidence has been mixed (personal communication).

The conclusions we reached are similar to findings from a qualitative secondary analysis conducted by Martin et al. [34] on evidence literacy in public health practice. They related to how public health practitioners define evidence, factors that influence what kind of evidence practitioners use in different contexts, and practitioner evidence preferences. This corroboration adds further weight to the connection between KT strategies and their impact on evidence use.

Further research is needed to explore how to make research evidence more accessible to front line staff. In addition, capacity building efforts for front line staff should include information on how to extrapolate findings from the literature to local context. By increasing the use of evidence in EFHIA, this study may support a normative change in the inclusion of equity within daily programmatic goals, through the development of EFHIA decision-support material. The learnings from this evaluation will enable health practitioners to respond to the World Health Organization call to action to reduce health inequities, as it has the potential to facilitate broader adoption of EFHIA across other Canadian provinces.

There were limitations in the data collected making it difficult to address all of our research questions regarding equity focused evidence use. While we hypothesized five initial propositions to test from our phase I research, our data points to only a few direct connections between mechanism and outcome, and they did not manifest exactly the way we hypothesized and defined the initial propositions. Our data only captured a small sample and may not reflect the entire organization’s perspectives. Staff changes on the EFHIA team also occurred near the end of the study, so not all the key informants and EFHIA team members interviewed at baseline and midpoint, respectively, were interviewed again at end of study. Because of this inconsistency, we were not able to capture all of the team members’ final perspectives. Another limitation is the varying amount of data collected from each study participant on each case study team. Furthermore, we found many interesting mechanisms occurring in single case study contexts, but were unable to extrapolate into semi-predictable patterns or demi-regularities. While we identified specific KT strategies that helped team members use evidence in the EFHIA tool, we were unable to make concrete connections between KT and evidence use. We had data that described specific evidence outcomes as well as the mechanisms that led to those outcomes, but had limited data that clearly illustrated the influence of KT strategies on mechanisms that triggered evidence use. Finally, information regarding organizational culture and policy was collected in 2016, and this context may have since changed and not be entirely reflective of the current health unit.

## Conclusion

The findings of this study provide a realist perspective on knowledge translation and evidence use in the implementation of EFHIA in public health practice. We identified important mechanisms, including evidence users’ intuitive appraisal of “return on investment” when using evidence and their preference to “identify with” evidence sources – leading to a preference for local and experience based data. Academic sources were less likely to have correspondence with users’ needs or consonance with the content. These findings can inform ongoing development and refinement of various knowledge translation interventions knowledge brokers could be used to mitigate these responses, with some limited success.

## Abbreviations

CMO: Context-Mechanism-Outcome
EFHIA: Equity Focused Health Impact Assessment
INSPQ: The Institut National de Santé Publique du Québec
KI: Key Informant
KT: Knowledge Translation
PHU: Public Health Unit
